# Epidemiology and patient journey of Rett syndrome in the United States: a real-world evidence study

**DOI:** 10.1186/s12883-023-03181-y

**Published:** 2023-04-04

**Authors:** Damian May, Kalé Kponee-Shovein, Malena Mahendran, Nathaniel Downes, Kristy Sheng, Patrick Lefebvre, Wendy Y. Cheng

**Affiliations:** 1 Acadia Pharmaceuticals Inc., 12830 El Camino Real, Ste. 400, San Diego, CA 92130 USA; 2 Analysis Group, Inc., 111 Huntington Avenue, 14th floor, Boston, MA 02199 USA

**Keywords:** Clinical manifestations, Costs, Economic burden, Healthcare resource utilization, Incidence, Prevalence, Epidemiology, Rett syndrome, Treatment patterns

## Abstract

**Background:**

Rett syndrome (RTT) is a neurodevelopmental disorder that almost exclusively affects females and is associated with high clinical burden. However, literature characterizing the real-world journey of patients with RTT is limited. This study provided an overview of the epidemiology, patient characteristics, clinical manifestations, healthcare resource utilization (HRU), costs, and treatment patterns of patients with RTT in the US.

**Methods:**

IQVIA™ Medical Claims Data and Longitudinal Prescription Data (11/01/2016–10/31/2019) were used to identify female patients with RTT, with the first observed diagnosis defined as the index date. Annual incidence and prevalence of RTT were assessed over the entire study period; clinical manifestations, all-cause and RTT-related HRU and costs, and treatment patterns were evaluated during the observation period—from the index date to end of clinical activity or end of data availability, whichever occurred first. Results were further stratified into pediatric (< 18 years) and adult (≥ 18 years) subgroups.

**Results:**

In 2019, prevalence and incidence of RTT was 0.32 and 0.23 per 10,000 enrollees, respectively. Among 5,940 female patients (pediatric: 3,078; adult: 2,862) with mean observation period of 2.04 years, the most prevalent clinical manifestations were neurological disorders (72.8%), gastrointestinal/nutritional disorders (41.9%), and orthopedic disorders (34.6%). The incidence rate of all-cause HRU was 44.43 visits per-patient-per-year and RTT-related HRU comprised 47% of all-cause HRU. Mean all-cause healthcare costs were $40,326 per-patient-per-year, with medical costs driven by home/hospice care visits, therapeutic services, outpatient visits, and inpatient visits. RTT-related healthcare costs comprised 45% of all-cause healthcare costs. The most prevalent supportive therapy and pharmacologic agent were feeding assistance (37.9%) and antiepileptic drugs (54.8%), respectively. Trends were similar by subgroup; although, rates of HRU were generally higher among pediatric patients relative to adult patients (all-cause: 52.43 and 35.86, respectively), which translated into higher mean healthcare costs (all-cause: $45,718 and $34,548, respectively).

**Conclusions:**

Patients with RTT have substantial disease burden, including prevalent clinical manifestations, high rates of HRU and annual healthcare costs, and reliance on pharmacologic and supportive therapies. These findings underscore the unmet need for effective therapies to target the multifactorial manifestations of RTT.

## Background

Rett syndrome (RTT) is a severe neurodevelopmental disorder that almost exclusively affects females, with an estimated incidence of 1 in 10,000 females by the age of 12 in the United States (US) [1, 2]. RTT is characterized by normal development during the first 6–18 months of life, followed by the progressive manifestation of key signs and symptoms, including loss of hand function skills and spoken language, gait abnormalities, and stereotyped hand movements [3, 4]. In most cases of classic RTT (90–95%), the disorder is caused by a spontaneous mutation in the *MECP2* gene on the X chromosome [3, 5]. While the increasing availability of genetic testing has facilitated diagnosis of RTT [3], there is often a delay between initial presentation and clinical diagnosis because of the heterogenous presentation of signs and symptoms among patients [6].

Due to the progressive nature of RTT, patients suffer from multisystem clinical manifestations, including neurological, gastrointestinal, cardiac, endocrine, and orthopedic disorders, that may evolve throughout the lifespan [7]. As such, lifelong care from multiple subspecialty providers is often required [3, 5]. However, to date, there is no cure for RTT, and current treatment options are aimed at managing symptoms and supporting activities of daily life [1, 8]. Consensus guidelines recommend early referral (i.e., from diagnosis to 5 years of age) to physical, occupational, and speech language therapists, as well as establishment of an individualized education program to promote childhood development [3]. Anticonvulsants may be used to treat seizures, while maintaining a healthy body mass and monitoring for scoliosis become important considerations as patients reach late childhood. Nevertheless, there are currently no approved therapies that target the underlying cause of RTT, highlighting an unmet need for effective, disease-modulating treatments.

In line with the high disease and increased care burden, RTT may be associated with substantial healthcare resource utilization (HRU) and costs [9]. A systematic literature review identified a few studies characterizing the economic burden of RTT in specific situations, such as respiratory-related hospital admissions or after surgical correction of scoliosis [9]. In a survey of 399 individuals with RTT, nearly one-quarter of respondents had a hospital admission for lower respiratory tract infection (LRTI) over the previous five years, highlighting the considerable respiratory morbidity of patients with RTT [10]. However, the literature remains scarce when evaluating the overall HRU and healthcare costs associated with RTT among patients in US clinical practice, representing a much-needed area of research.

In light of the gaps in the literature regarding RTT, this study aimed to provide an overview of the epidemiology, patient characteristics, clinical manifestations, HRU, costs, and treatment patterns of patients with RTT in the US.

## Methods

### Data source

Administrative healthcare claims data from the IQVIA™ Medical Claims Data (Dx) and Longitudinal Prescription Data (LRx) databases (11/1/2016–10/31/2019) were used to address the study objectives. IQVIA Dx and LRx databases are large, de-identified, open administrative claims databases containing healthcare claims for over 130 million beneficiaries. The IQVIA Dx database includes pre-adjudicated medical claims collected from office-based physicians and specialists; while the IQVIA LRx database includes adjudicated, prescription claims collected from transactional records and key pharmacy locations. Both databases comprise retrospective information on patient demographics, patient-level diagnoses, procedures, and in-office treatments, as well as retail, mail, and long-term care facility pharmacy data. Diagnoses and procedures based on International Classification of Diseases, 10^th^ Revision, Clinical Modification (ICD-10-CM) and Current Procedural Terminology, 4^th^ edition codes are included.

Data were de-identified and compliant with the Health Insurance Portability and Accountability Act; therefore, no institutional review board reviews were required.

### Study design

A longitudinal, retrospective, cohort study design was used. The index date was defined as the date of the first observed medical claim with a diagnosis of RTT. The baseline period comprised the period up to 6 months prior to the index date for patients aged ≥ 1 year, or the period from the start of clinical activity to the index date for patients aged < 1 year. The observation period was defined as the period from the index date to the earliest of end of clinical activity (i.e., last available medical or pharmacy claim) or end of data availability.

### Study population

For the analysis of prevalence and incidence of RTT, all male and female enrollees were included. For the remaining analyses, female patients with at least one medical claim with a diagnosis code of RTT (ICD-10-CM: F84.2) in any position and up to 6 months of clinical activity prior to the index date were included in the study. A diagnosis code of RTT in any position was considered for the current study to capture the complete burden of RTT. Additionally, male patients with RTT were excluded because of uncertainty in correctly identifying these patients with high specificity based on exploratory investigations of the data. Patients with medical claims with a primary or secondary diagnosis code for cerebrovascular disease or brain trauma during the baseline period were excluded from the study population. Patients were further stratified by age on the index date into pediatric (< 18 years) and adult (≥ 18 years) subgroups.

### Study outcomes

The annual incidence and prevalence of RTT were assessed over the entire study period. Outcomes measured during the observation period included frequency of common clinical manifestations of RTT, all-cause and RTT-related HRU and costs, as well as treatment patterns.

Clinical manifestations included neurological disorders, gastrointestinal and nutritional disorders, orthopedic disorders, oral disorders, endocrine disorders, and prolonged QT interval; events were identified as any day on which a medical claim with a diagnosis of a clinical manifestation was observed in any position.

All-cause and RTT-related HRU and pre-adjudicated medical costs included those for inpatient, emergency department (ED), outpatient (OP, i.e., office, clinic, or hospital OP), long-term care/skilled nursing facility, other place of service (i.e., home/hospice, independent laboratory, hospital laboratory services, ambulance, telehealth, among others), and unspecified place of service. Adjudicated pharmacy costs were also reported. RTT-related HRU and medical costs were defined as those associated with any medical service claim with a diagnosis of RTT in any position, while RTT-related pharmacy costs were defined as all costs associated with pharmacy claims for RTT-related therapy (i.e., antiepileptics, nutritional agents, sedatives, prokinetic agents, and antiarrhythmics).

Treatment patterns included the use of supportive therapy (i.e., feeding assistance, other home/hospice care, physical therapy, speech-language therapy, occupational therapy, scoliosis surgery, hydrotherapy) and pharmacologic agents (i.e., antiepileptic drugs, sedative/hypnotics, prokinetic agents, nutritional supplements, antiarrhythmic drugs) during the observation period.

### Statistical analysis

All statistical analyses were conducted using SAS Enterprise Guide Version 7.1 (SAS Institute, Cary, NC, USA). Annual incidence was calculated as the proportion of patients newly diagnosed with RTT among the total number of at-risk patients at the start of the study period. A minimum 6-month washout period was used to ensure at-risk enrollees and incident cases did not have a RTT diagnosis. Annual prevalence was calculated as the proportion of patients with a diagnosis of RTT among the total number of patients enrolled in the IQVIA Dx and LRx databases.

Baseline patient characteristics, clinical manifestations, HRU, costs, and treatment patterns were described for the overall RTT study population and by pediatric and adult subgroups. Continuous characteristics were summarized using mean, standard deviation (SD), and median values, while categorical characteristics were summarized using relative frequencies and proportions.

Clinical manifestations were also described by age on the index date as the number of clinical manifestation events and the proportion of patients with ≥ 1 clinical manifestation per year using a panel plot. Similarly, treatment patterns were described by age on the index date as the proportion of patients using supportive therapy or pharmacologic agents per year using a panel plot.

To account for varying lengths of follow-up, annual incidence rates of HRU and mean healthcare costs were reported per-patient-per-year (PPPY). All costs were inflation-adjusted to 2021 US dollars (USD).

Extreme values were truncated at the 95^th^ percentile for panel plots and healthcare costs to reduce sensitivity to outliers.

## Results

### Prevalence and incidence of RTT

The annual prevalence of RTT between November 1, 2016 and October 31, 2019 in this dataset ranged from 0.30–0.32 per 10,000 enrollees overall, with higher prevalence ranges observed among female than male patients (female: 0.45–0.52; male: 0.08–0.10; Table 1).

**Table 1:** Annual Prevalence of RTT among Enrollees between November 1, 2016 and October 31, 2019,^a^ Overall and by Sex

Prevalence estimate	Overall^b^			Female			Male		
	**2017**	**2018**	**2019**	**2017**	**2018**	**2019**	**2017**	**2018**	**2019**
Total enrollees^c^	160,281,754	160,528,443	151,839,417	90,690,442	89,701,300	82,296,964	69,591,312	70,827,143	69,542,453
Prevalent RTT cases^d^	4,764	4,982	4,852	4,125	4,295	4,285	638	685	566
Annual prevalence estimate per 10,000 enrollees	0.30	0.31	0.32	0.45	0.48	0.52	0.09	0.10	0.08

*Abbreviations*: *RTT* Rett Syndrome, *ICD-10-CM* International Classification of Diseases, 10th Revision, Clinical Modification

^a^Individual years were defined as starting from November 1 of the prior year to October 31 of the year described. For example, year 2017 begins on November 1, 2016 and ends on October 31, 2017

^b^Includes enrollees identified as female, male, and of unknown sex

^c^Total number of enrollees in the IQVIA database was obtained from IQVIA directly

^d^Patients with ≥ 1 medical claim with a diagnosis of RTT during each year of interest. RTT was identified using the following ICD-10-CM diagnosis codes in any position: F84.2

Overall, the annual incidence of RTT in this dataset was an estimated 0.34 and 0.23 per 10,000 enrollees in 2018 and 2019, respectively (Table 2). Among female patients, the annual incidence was 0.43 and 0.31 per 10,000 female enrollees in 2018 and 2019, respectively, while among male patients, it was 0.22 and 0.13 per 10,000 male enrollees, respectively. Stratified by age, incidence estimates per 10,000 enrollees for the pediatric population in 2018 and 2019 were 1.40 and 1.25, respectively, for ages 0–4 years; 1.53 and 0.92 for ages 5–10 years; and 1.00 and 0.67 for ages 11–17 years (Table 3).

**Table 2:** Annual Incidence of RTT among Enrollees between November 1, 2017 and October 31, 2019,^a^ Overall and by Sex

Incidence estimate^b^	Overall^c^		Female		Male	
	**2018**	**2019**	**2018**	**2019**	**2018**	**2019**
At-risk enrollees^d^	34,336,947	35,994,590	19,567,060	20,439,789	14,760,187	15,544,934
Incident RTT cases^e^	1,167	840	840	631	327	209
Annual incidence proportion estimate per 10,000 enrollees	0.34	0.23	0.43	0.31	0.22	0.13

*Abbreviations*: *RTT* Rett Syndrome, *ICD-10-CM* International Classification of Diseases, 10th Revision, Clinical Modification

^a^The study period spanned from November 1, 2017 to October 31, 2019 for the incidence estimation, as data from the time prior served as a washout period to define the at-risk population and exclude prevalent patients with RTT. Individual years were defined as starting from November 1 of the prior year to October 31 of the year described. For example, year 2018 begins on November 1, 2017 and ends on October 31, 2018

^b^A minimum 6-month washout period was used to ensure at-risk enrollees and incident cases did not have any RTT diagnosis ≥ 6 months prior to the start of the study period (November 1 of each year), extending to the start of clinical activity for each enrollee

^c^Includes enrollees identified as female, male, and of unknown sex

^d^At-risk enrollees were identified as enrollees with clinical activity at the start of the study period (November 1 of each year of interest) and with no prior diagnosis of RTT during the washout period. The at-risk enrollee count was calculated by subtracting the number of enrollees with a diagnosis of RTT from the total number of enrollees in the IQVIA database in each given year

^e^Patients with ≥ 1 medical claim with a diagnosis of RTT and no prior diagnosis code for RTT during the washout period were considered as incident cases. RTT was identified using the following ICD-10-CM diagnosis codes in any position: F84.2

**Table 3:** Annual Incidence of RTT Among Enrollees between November 1, 2017 and October 31, 2019,^a^ Overall and Stratified by Age

Incidence estimate^b^	Overall^c^		Age 0–4		Age 5–10		Age 11–17	
	**2018**	**2019**	**2018**	**2019**	**2018**	**2019**	**2018**	**2019**
At-risk enrollees^d^	34,336,947	35,994,590	1,669,754	1,602,333	1,438,340	1,587,001	1,765,739	1,917,644
Incident RTT cases^e^	1,167	840	233	200	220	146	177	128
Annual incidence proportion estimate per 10,000 enrollees	0.34	0.23	1.40	1.25	1.53	0.92	1.00	0.67
**Incidence estimate**^**b**^	**Age 18–29**		**Age 30–39**		**Age 40–49**		**Age 50 + **	
	**2018**	**2019**	**2018**	**2019**	**2018**	**2019**	**2018**	**2019**
At-risk enrollees^d^	2,429,615	2,883,526	2,749,971	2,976,747	3,496,970	3,535,645	20,786,558	21,491,694
Incident RTT cases^e^	239	140	126	95	58	44	83	67
Annual incidence proportion estimate per 10,000 enrollees	0.98	0.49	0.46	0.32	0.17	0.12	0.04	0.03

*Abbreviations*: *RTT* Rett Syndrome, *ICD-10-CM* International Classification of Diseases, 10th Revision, Clinical Modification

^a^The study period spanned from November 1, 2017 to October 31, 2019 for the incidence estimation, as data from the time prior served as a washout period to define the at-risk population and exclude prevalent patients with RTT. Individual years were defined as starting from November 1 of the prior year to October 31 of the year described. For example, year 2018 begins on November 1, 2017 and ends on October 31, 2018

^b^A minimum 6-month washout period was used to ensure at-risk enrollees and incident cases did not have any RTT diagnosis ≥ 6 months prior to the start of the study period (November 1 of each year), extending to the start of clinical activity for each enrollee

^c^Includes enrollees identified as female, male, and of unknown sex

^d^At-risk enrollees were identified as enrollees with clinical activity at the start of the study period (November 1 of each year of interest) and with no prior diagnosis of RTT during the washout period. The at-risk enrollee count was calculated by subtracting the number of enrollees with a diagnosis of RTT from the total number of enrollees in the IQVIA database in each given year

^e^Patients with ≥ 1 medical claim with a diagnosis of RTT and no prior diagnosis code for RTT during the washout period were considered as incident cases. RTT was identified using the following ICD-10-CM diagnosis codes in any position: F84.2

### Study population, baseline demographics, and clinical characteristics

**Fig. 1:**
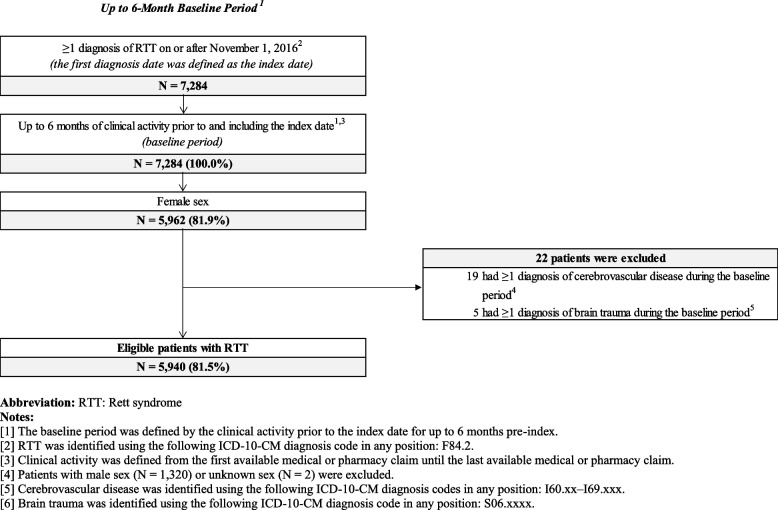
Patient Disposition

There were 5,940 female patients included in the study population after all eligibility criteria were applied; 3,078 (52%) comprised the pediatric cohort and 2,862 (48%) comprised the adult cohort (Fig. 1).

The mean age of the female population with RTT was 20.0 years and frequency of *MECP2* genetic testing prior to the index RTT diagnosis was low (1.2%; Table 4). Among known rendering provider specialties, genetic tests were most often administered by a pediatrician or pediatric specialist (23.2%). Overall, 16.2% of patients (pediatric: 19.9%; adult: 12.2%) had ≥ 1 differential diagnosis prior to their index RTT diagnosis, most often as cerebral palsy, autism spectrum disorder, or non-specific developmental delay. All-cause HRU during the baseline period among female patients with RTT was 3.01 visits per person-month, which was primarily driven by OP visits, other places of service, and home/hospice care.

**Table 4:** Baseline Demographics and Clinical Characteristics among Patients with RTT, Overall and by Pediatric and Adult Patients

Characteristics	Overall RTT cohort	Stratification by Age	
		**Pediatric** **(< 18 years of age)**	**Adult** **(≥ 18 years of age)**
	***N* = 5,940**	***N* = 3,078**	***N* = 2,862**
**Demographics**^a^			
Age at index date, years, mean ± SD [median]	20.0 ± 14.5 [17.0]	9.2 ± 4.6 [9.0]	31.6 ± 12.5 [29.0]
Pediatric, n (%)			–
0–4	612 (10.3)	612 (19.9)	–
5–10	1,194 (20.1)	1,194 (38.8)	–
11–17	1,272 (21.4)	1,272 (41.3)	–
Adult, n (%)			
18–29	1,513 (25.5)	–	1,513 (52.9)
30–39	786 (13.2)	–	786 (27.5)
40–49	316 (5.3)	–	316 (11.0)
≥ 50	247 (4.2)	–	247 (8.6)
Region, n (%)			
South	2,051 (34.5)	1,155 (37.5)	896 (31.3)
West	1,373 (23.1)	716 (23.3)	657 (23.0)
Midwest	1,328 (22.4)	648 (21.1)	680 (23.8)
Northeast	1,151 (19.4)	538 (17.5)	613 (21.4)
Other^b^	4 (0.1)	2 (0.1)	2 (0.1)
Unknown/Unspecified	33 (0.6)	19 (0.6)	14 (0.5)
Insurance plan type at index date, n (%)			
Unknown/Unspecified Plan^c^	1,658 (27.9)	998 (32.4)	660 (23.1)
Medicaid	1,621 (27.3)	858 (27.9)	763 (26.7)
Commercial	1,101 (18.5)	675 (21.9)	426 (14.9)
Medicare/Medicaid Dual Eligible	895 (15.1)	528 (17.2)	367 (12.8)
Medicare	665 (11.2)	19 (0.6)	646 (22.6)
**Year of index date, n (%)**			
2016	2,224 (37.4)	1,203 (39.1)	1,021 (35.7)
2017	2,095 (35.3)	1,024 (33.3)	1,071 (37.4)
2018	1,014 (17.1)	522 (17.0)	492 (17.2)
2019	607 (10.2)	329 (10.7)	278 (9.7)
**Quan-CCI,**^d**,**e^** mean ± SD [median]**	0.1 ± 0.4 [0.0]	0.1 ± 0.3 [0.0]	0.1 ± 0.5 [0.0]
***MECP2* genetic testing,** ^d ,f^** n (%)**	69 (1.2)	61 (2.0)	8 (0.3)
Time from* MECP2 *genetic testing date to index date,^g^ days, mean ± SD [median]	51.5 ± 46.0 [45.0]	57.2 ± 45.3 [53.0]	8.1 ± 23.0 [0.0]
*Rendering provider specialty,* ^d*,* g* ,*h^* n (%)*			
Unknown/missing	41 (59.4)	37 (60.7)	4 (50.0)
Pediatrician/pediatric specialist^i^	16 (23.2)	14 (23.0)	2 (25.0)
Genetics/pathology specialist	7 (10.1)	7 (11.5)	0 (0.0)
Primary care	4 (5.8)	2 (3.3)	2 (25.0)
Neurologist	1 (1.4)	1 (1.6)	0 (0.0)
**Differential diagnosis of RTT,** ^d^** n (%)**			
*Any differential diagnosis*	963 (16.2)	613 (19.9)	350 (12.2)
Autism spectrum disorder	367 (6.2)	269 (8.7)	98 (3.4)
Cerebral palsy	413 (7.0)	171 (5.6)	242 (8.5)
Non-specific developmental delay	321 (5.4)	284 (9.2)	37 (1.3)
Angelman syndrome	2 (0.0)	2 (0.1)	0 (0.0)
Other childhood disintegrative disorder	6 (0.1)	3 (0.1)	3 (0.1)
**All-cause healthcare resource utilization,**^**j–l**^** PPM, mean ± SD [median]**	3.01 ± 5.92 [0.94]	3.56 ± 6.34 [1.05]	2.46 ± 5.42 [0.85]
***By place of service***^***m***^			
Inpatient stay	0.08 ± 1.17 [0.00]	0.05 ± 0.93 [0.00]	0.10 ± 1.36 [0.00]
ED visit	0.07 ± 0.68 [0.00]	0.07 ± 0.55 [0.00]	0.07 ± 0.78 [0.00]
OP visit	0.79 ± 2.44 [0.00]	1.08 ± 2.83 [0.17]	0.50 ± 1.93 [0.00]
Long-term care/skilled nursing facilities	0.02 ± 0.29 [0.00]	0.00 ± 0.02 [0.00]	0.03 ± 0.41 [0.00]
Other place of service^n^	0.88 ± 3.88 [0.00]	0.91 ± 4.05 [0.00]	0.85 ± 3.70 [0.00]
*Unknown place of service*^o^	1.17 ± 3.73 [0.00]	1.44 ± 4.15 [0.00]	0.90 ± 3.23 [0.00]
Home/hospice care	0.36 ± 2.55 [0.00]	0.44 ± 2.86 [0.00]	0.28 ± 2.18 [0.00]
Therapeutic services visit^p^	0.32 ± 1.62 [0.00]	0.46 ± 1.85 [0.00]	0.18 ± 1.34 [0.00]
Medical supplies^q^	0.21 ± 1.10 [0.00]	0.18 ± 1.05 [0.00]	0.25 ± 1.16 [0.00]
Durable medical equipment use^r^	0.08 ± 0.76 [0.00]	0.10 ± 0.89 [0.00]	0.06 ± 0.60 [0.00]
Other^s^	0.22 ± 1.36 [0.00]	0.33 ± 1.70 [0.00]	0.10 ± 0.89 [0.00]

*Abbreviations*: *CPT* Current Procedural Terminology, *ED* Emergency department, *HCPCS* Healthcare Common Procedure Coding System, *OP* Outpatient, *PPM* Per person-month, *Quan-CCI* Quan-Charlson comorbidity index, *RTT* Rett syndrome, *SD* Standard deviation

^a^Evaluated on the index date (i.e., date of the first observed medical claim with a diagnosis of RTT)

^b^Includes Puerto Rico, Virgin Islands and Guam

^c^Includes medical claims associated with an unspecified plan, unknown third party, cash, claims processing, or missing

^d^Evaluated on each distinct day during the baseline period and including the index date

^e^Reference: Quan, H., et al. (2005). Coding algorithms for defining comorbidities in ICD-9-CM and ICD-10 administrative data. Medical Care, 43(11): 1130–39

^f^Identified using CPT codes: 81,302–81,304, 0234U, 81,470, 81,471, 81,479

^g^*MECP2* genetic testing date was based on the medical claim associated with a procedure code for *MECP2* genetic testing closest to the index date and including on the index date

^h^Based on the specialty of the provider who rendered the service of medical claims associated with a procedure code for *MECP2* genetic testing

^i^Pediatric specialist included child neurology, developmental/behavioral pediatrics, pediatric cardiology, pediatric endocrinology, pediatric gastroenterology, and pediatric radiology

^j^Among patients with multiple types of visits on the same day, inpatient stays were prioritized over all other types of visits, followed by ED visits, OP visits, long-term care/skilled nursing facilities, other places of service, and unknown place of service

^k^Consecutive days of inpatient stays, ED visits, or long-term care visits were considered one visit

^l^Evaluated on each distinct day during the baseline period, not including the index date, among patients with ≥ 1 day of clinical activity prior to the index date (N = 4,399)

^m^Place of service was defined by the place associated with each medical claim for which a patient sought healthcare services

^n^Other places of service included home/hospice, independent laboratory, hospital laboratory services provided to non-patients, ambulance, telehealth, and more

^o^Unknown places of service included medical claims associated with other places of service or unassigned places of service. Procedure codes associated with each medical claim were used to define the type of visit

^p^Therapeutic services visit included physical therapy, hydrotherapy, occupational therapy, speech therapy, and feeding assistance

^q^Defined as any medical claim with a procedure code for medical supplies. Medical supplies were identified using the following HCPCS procedure codes: A4xxx–A9xxx and T4xxx–T5xxx

^r^Defined as visits that include at least one medical claim with a procedure code for DME. DME was identified using the following HCPCS procedure codes: E0xxx–E8xxx

^s^Defined as any medical claims with a procedure code not captured under home/hospice care, therapeutic services, medical supplies, or durable medical equipment. Commonly observed procedure codes included educational habilitation, therapeutic behavioral services, adaptive behavior therapy by protocol, community-based wrap-around services, and day habilitation waiver

### Frequency of common clinical manifestations of RTT

**Fig. 2:**
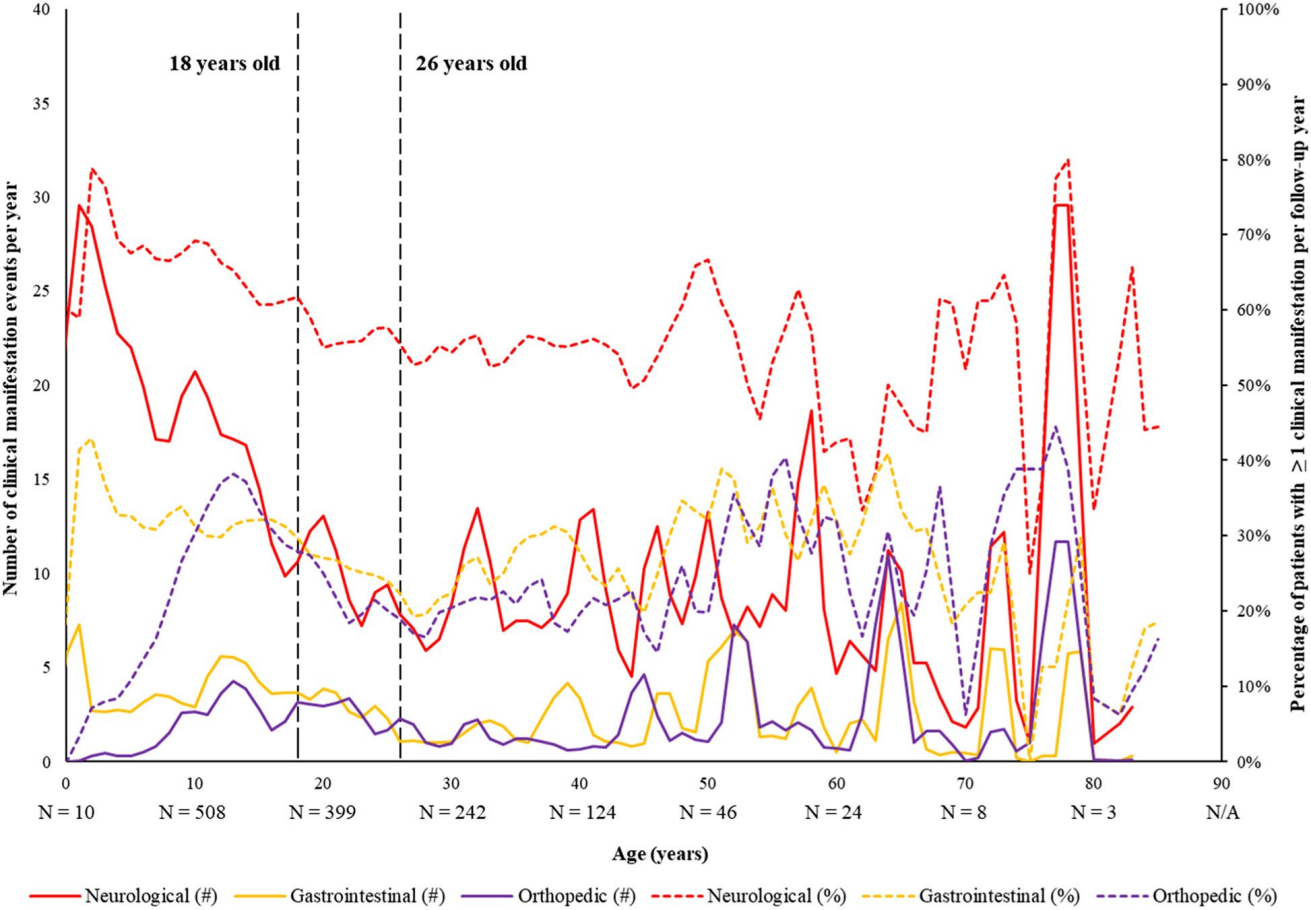
Average Number and Percentage of Yearly Clinical Manifestations by Age During the Observation Period, All Patients

Over a mean observation period of 2.04 years, the most prevalent clinical manifestations among female patients with RTT were neurological disorders (72.8%), primarily driven by epilepsy (52.1%), followed by gastrointestinal and nutritional disorders (41.9%) and orthopedic disorders (34.6%; Table 5). Based on the panel plot data (Fig. 2), the prevalence and number of neurological manifestation events were highest in early childhood (1–3 years of age) before decreasing and reaching a plateau into early adulthood (~ 26 years of age). Similarly, the number of gastrointestinal manifestation events were highest in early childhood (2 years of age) before plateauing into adulthood and increasing slightly into late adulthood (50 years of age and onwards).

**Table 5:** Frequency of Clinical Manifestations among Patients with RTT, Overall and by Pediatric and Adult Patients

Clinical manifestations^a,b^	Patients with ≥ 1 Clinical Manifestation (%)		
	**Overall RTT cohort**	**Stratification by Age**	
		**Pediatric** **(< 18 years of age)**	**Adult** **(≥ 18 years of age)**
	***N* = 5,940**	***N* = 3,078**	***N* = 2,862**
**Observation period,**^c^** years, mean ± SD [median]**	2.04 ± 0.96 [2.4]	2.04 ± 0.98 [2.4]	2.04 ± 0.95 [2.4]
**Total person-years**	12,111	6,264	5,847
Neurological disorders	4,323 (72.8)	2,366 (76.9)	1,957 (68.4)
Epilepsy	3,094 (52.1)	1,570 (51.0)	1,524 (53.2)
Dysphagia	1,395 (23.5)	753 (24.5)	642 (22.4)
Loss of acquired communication skills	859 (14.5)	724 (23.5)	135 (4.7)
Behavioral disorders and disturbance symptoms	650 (10.9)	392 (12.7)	258 (9.0)
Gastrointestinal and nutritional disorders	2,489 (41.9)	1,373 (44.6)	1,116 (39.0)
Constipation	1,543 (26.0)	852 (27.7)	691 (24.1)
Gastroesophageal reflux	1,079 (18.2)	593 (19.3)	486 (17.0)
Vomiting/regurgitation	636 (10.7)	395 (12.8)	241 (8.4)
Orthopedic disorders	2,054 (34.6)	1,118 (36.3)	936 (32.7)
Scoliosis	1,633 (27.5)	997 (32.4)	636 (22.2)
Oral disorders^d^	582 (9.8)	287 (9.3)	295 (10.3)
Endocrine disorders	312 (5.3)	88 (2.9)	224 (7.8)
Prolonged QT interval	132 (2.2)	94 (3.1)	38 (1.3)

*Abbreviations*: *RTT* Rett syndrome, *SD* Standard deviation

^a^Clinical manifestation events were defined as any day during which an ICD-10-CM code for a clinical manifestation was observed. Incidence rates were calculated as the total number of clinical manifestation events divided by the total number of person-years for the respective cohort

^b^Only conditions with prevalence ≥ 10% are shown per category

^c^The observation period was defined as the period from the index date (i.e., date of the first observed medical claim with a diagnosis of RTT) to the earliest of end of clinical activity or end of data availability (i.e., October 31, 2019)

^d^Frequency of oral disorders were evaluated using medical benefit claims only and may be underestimated if patients had third-party dental insurance

### All-cause and RTT-related HRU

The incidence rate of all-cause HRU by place of service among female patients with RTT was 44.43 visits PPPY during the observation period, primarily driven by home/hospice care (16.31 visits PPPY), OP visits (9.58 visits PPPY), and therapeutic service visits (7.26 visits PPPY; Table 6). All-cause HRU largely comprised of RTT-related HRU, with 47% of the all-cause incidence rate attributed to a RTT diagnosis in the overall study population (Table 6). A higher incidence rate of RTT-related HRU was observed in the pediatric subgroup relative to the adult subgroup across all types of HRU, excluding long-term care/skilled nursing facilities.

**Table 6:** HRU among Patients with RTT, Overall and by Pediatric and Adult Patients

Resource utilization^a–c^	Incidence rate (PPPY)		
	**Overall RTT cohort**	**Stratification by Age**	
		**Pediatric (< 18 years of age)**	**Adult (≥ 18 years of age)**
	***N*** **= 5,940**	***N*** **= 3,078**	***N*** **= 2,862**
**Observation period,**^d^** years, mean ± SD [median]**	2.04 ± 0.96 [2.42]	2.04 ± 0.98 [2.42]	2.04 ± 0.95 [2.42]
**Total person-years**	12,111	6,264	5,847
**All-cause HRU**	44.43	52.43	35.86
Inpatient stay	0.37	0.36	0.38
ED visit	0.56	0.56	0.57
OP visit	9.58	13.22	5.69
Long-term care/skilled nursing facilities	0.17	0.02	0.32
Other place of service^e^	2.30	2.29	2.30
*Unknown place of service*^f^	31.46	35.97	26.61
Home/hospice care	16.31	18.50	13.96
Therapeutic services visit^g^	7.26	9.57	4.78
Medical supplies^h^	5.41	5.25	5.59
Durable medical equipment use^i^	2.47	2.99	1.91
Other^j^	2.81	4.11	1.41
**RTT-related HRU**^k^	20.98	25.14	16.52
Inpatient stay	0.21	0.24	0.17
ED visit	0.12	0.13	0.11
OP visit	4.39	6.53	2.10
Long-term care/skilled nursing facilities	0.07	0.02	0.14
Other place of service^e^	1.04	1.14	0.93
*Unknown place of service*^f^	15.14	17.09	13.07
Home/hospice care	7.57	7.95	7.16
Therapeutic services visit^g^	3.83	5.16	2.41
Medical supplies^h^	2.43	2.54	2.30
Durable medical equipment use^i^	1.21	1.48	0.93
Other^j^	1.39	2.10	0.62

*Abbreviations*: *CPT* Current Procedural Terminology, *ED* Emergency department, *HCPCS* Healthcare Common Procedure Coding System, *HRU* Healthcare resource utilization, *OP* Outpatient, *PPPY* Per-patient-per-year, *RTT* Rett syndrome, *SD* Standard deviation

^a^Among patients with multiple types of visits on the same day, inpatient stays were prioritized over all other types of visits, followed by ED visits, OP visits, long-term care/skilled nursing facilities, other places of service, and unknown place of service

^b^Consecutive days of inpatient stays, ED visits, or long-term care visits were considered one visit

^c^Evaluated on each distinct day during the observation period, including the index date

^d^The observation period was defined as the period from the index date (i.e., date of the first observed medical claim with a diagnosis of RTT) to the earliest of end of clinical activity or end of data availability (i.e., October 31, 2019)

^e^Other places of service included home/hospice, independent laboratory, hospital laboratory services provided to non-patients, ambulance, telehealth, and more

^f^Unknown places of service included medical claims associated with other places of service or unassigned places of service. Procedure codes associated with each medical claim were used to define the type of visit

^g^Therapeutic services visit included physical therapy, hydrotherapy, occupational therapy, speech therapy, and feeding assistance

^h^Defined as any medical claim with a procedure code for medical supplies. Medical supplies were identified using the following HCPCS procedure codes: A4xxx–A9xxx and T4xxx–T5xxx

^i^Defined as visits that include at least one medical claim with a procedure code for DME. DME was identified using the following HCPCS procedure codes: E0xxx–E8xxx

^j^Defined as any medical claims with a procedure code not captured under home/hospice care, therapeutic services, medical supplies, or durable medical equipment. Commonly observed procedure codes included comprehensive community support services, waiver services (not otherwise specified), educational habilitation, intensive, and extended multidisciplinary services provided in a clinic setting, and non-emergency transportation

^k^RTT-related was defined as a medical service claim with a diagnosis of RTT in the primary or secondary position. RTT was identified using the following ICD-10-CM diagnosis code: F84.2

### All-cause and RTT-related healthcare costs

During the observation period, female patients with RTT incurred mean all-cause total healthcare costs of $34,772 (86%) in pre-adjudicated medical costs and $5,554 (14%) in adjudicated pharmacy costs (Table 7). Mean all-cause medical costs were primarily driven by home/hospice care visits ($12,054), followed by therapeutic services ($7,071), OP visits ($6,791), and inpatient visits ($6,088) among the overall cohort.

**Table 7:** Healthcare Costs among Patients with RTT, Overall and by Pediatric and Adult Patients

PPPY healthcare costs,^a–b^ $US 2021	Overall RTT cohort	Stratification by Age	
		**Pediatric (< 18 years of age)**	**Adult (≥ 18 years of age)**
	***N* = 5,940**	***N* = 3,078**	***N* = 2,862**
**All-cause total healthcare costs, PPPY, mean ± SD [median]**			
Medical costs	34,772 ± 42,747 [16,605]	40,258 ± 45,023 [22,397]	28,893 ± 39,324 [11,705]
Inpatient	6,088 ± 15,776 [0]	6,718 ± 16,482 [0]	5,412 ± 14,954 [0]
ED	961 ± 1,911 [0]	951 ± 1,883 [0]	972 ± 1,941 [0]
OP	6,791 ± 10,012 [2,269]	8,379 ± 10,972 [3,611]	5,088 ± 8,548 [1,387]
Long-term care/skilled nursing facilities^c^	0 ± 0 [0]	0 ± 0 [0]	0 ± 0 [0]
Other place of service	1,063 ± 2,041 [61]	1,141 ± 2,134 [26]	980 ± 1,932 [84]
*Unknown place of service*^d^	19,870 ± 31,660 [5,929]	23,069 ± 33,274 [8,105]	16,440 ± 29,451 [3,922]
Home/hospice care	12,054 ± 58,527 [0]	15,161 ± 66,024 [0]	8,725 ± 49,022 [0]
Therapeutic services^e^	7,071 ± 19,386 [0]	7,802 ± 17,538 [193]	6,287 ± 21,161 [0]
Medical supplies	1,712 ± 4,432 [595]	1,738 ± 3,602 [596]	1,685 ± 5,175 [595]
Durable medical equipment	2,942 ± 12,096 [93]	3,925 ± 13,321 [631]	1,889 ± 10,526 [0]
Other/missing	3,491 ± 24,612 [259]	3,654 ± 24,535 [624]	3,317 ± 24,692 [68]
Pharmacy costs	5,554 ± 8,958 [1,146]	5,459 ± 8,941 [1,046]	5,655 ± 8,975 [1,253]
**RTT-related total healthcare costs,**^f^** PPPY, mean ± SD [median]**			
Medical costs	14,643 ± 20,160 [5,908]	17,343 ± 21,045 [8,969]	11,748 ± 18,739 [3,463]
Inpatient	2,235 ± 6,294 [0]	2,656 ± 6,804 [0]	1,784 ± 5,663 [0]
ED	52 ± 169 [0]	58 ± 177 [0]	45 ± 161 [0]
OP	2,692 ± 4,778 [475]	3,513 ± 5,358 [857]	1,813 ± 3,878 [279]
Long-term care/skilled nursing facilities^c^	0 ± 0 [0]	0 ± 0 [0]	0 ± 0 [0]
Other place of service	425 ± 965 [0]	498 ± 1,055 [0]	348 ± 851 [0]
*Unknown place of service*^d^	9,238 ± 16,346 [2,148]	10,618 ± 16,947 [3,334]	7,759 ± 15,541 [1,144]
Home/hospice care	5,026 ± 30,728 [0]	5,464 ± 28,617 [0]	4,557 ± 32,832 [0]
Therapeutic services^e^	3,973 ± 16,395 [0]	4,054 ± 13,873 [0]	3,887 ± 18,725 [0]
Medical supplies	770 ± 1,998 [0]	833 ± 2,136 [0]	703 ± 1,836 [0]
Durable medical equipment	1,949 ± 11,048 [0]	2,624 ± 12,130 [0]	1,226 ± 9,704 [0]
Other/missing	1,578 ± 12,212 [0]	1,581 ± 11,105 [92]	1,575 ± 13,296 [0]
Pharmacy costs	3,428 ± 6,761 [223]	3,433 ± 6,770 [216]	3,422 ± 6,753 [230]

*Abbreviations*: *ED* Emergency department, *OP* Outpatient, *PPPY* Per-patient-per-year, *RTT* Rett syndrome, *SD* Standard deviation, *$US* United States Dollar

^a^Among patients with multiple types of visits on the same day, inpatient stays were prioritized over all other types of visits, followed by ED visits, OP visits, long-term care/skilled nursing facilities, other places of service, and unknown place of service

^b^All costs were truncated at the 95th percentile. For patients with no utilization of medical or pharmaceutical services, costs were set to zero dollars

^c^Medical claims with non-zero costs for long-term care/skilled nursing facility fell above the 95th percentile of costs among patients with RTT

^d^Unknown places of service included medical claims that described the place of service as “other” or “unassigned”. Procedure codes associated with each medical claim were used to define the type of visit

^e^Therapeutic services visit included physical therapy, hydrotherapy, occupational therapy, speech therapy, and feeding assistance

^f^RTT-related medical costs were defined as all costs for a medical service claim with a diagnosis of RTT in the primary or secondary position. RTT was identified using the following ICD-10-CM diagnosis code: F84.2. RTT-related pharmacy costs were defined as all costs for a pharmacy claim for an RTT-related therapy (i.e., antiepileptics, nutritional agents, sedatives, prokinetic agents, and antiarrhythmics)

Mean RTT-related total healthcare costs PPPY included $14,643 in pre-adjudicated medical costs and $3,428 in adjudicated pharmacy costs (Table 7). Mean RTT-related pre-adjudicated medical costs accounted for 42% of all-cause pre-adjudicated medical costs, while RTT-related pharmacy costs accounted for 62% of all-cause pharmacy costs. As with all-cause medical costs, key drivers of RTT-related medical costs included home/hospice care visits ($5,026), therapeutic services ($3,973), OP visits ($2,692), and inpatient visits ($2,235).

Mean healthcare costs were generally higher in the pediatric subgroup relative to the adult subgroup, with similar trends to the overall population observed in the breakdown of cost components (Table 7).

### Treatment patterns

Among supportive therapies used, feeding assistance was the most prevalent (37.9%), followed by other home/hospice care (24.9%) and physical therapy (24.4%; Table 8). Additionally, antiepileptic drugs were the most prevalent pharmacologic therapy used (54.8%; Table 8).

**Table 8:** Treatment Patterns During the Observation Period among Patients with RTT, Overall and by Pediatric and Adult Patients

Treatment Patterns	Overall RTT cohort	Stratification by Age	
		**Pediatric (< 18 years of age)**	**Adult (≥ 18 years of age)**
	***N* = 5,940**	***N* = 3,078**	***N* = 2,862**
**Observation period,**^**a**^** years, mean ± SD [median]**	2.04 ± 0.96 [2.42]	2.04 ± 0.98 [2.42]	2.04 ± 0.95 [2.42]
**Supportive therapy**			
***Patients with* ≥ *1 therapy, n (%)***	3,523 (59.3)	2,042 (66.3)	1,481 (51.7)
Feeding assistance	2,253 (37.9)	1,332 (43.3)	921 (32.2)
Other home/hospice care	1,482 (24.9)	765 (24.9)	717 (25.1)
Physical therapy	1,450 (24.4)	1,024 (33.3)	426 (14.9)
Speech-language therapy	791 (13.3)	668 (21.7)	123 (4.3)
Occupational therapy	681 (11.5)	508 (16.5)	173 (6.0)
Scoliosis surgery	70 (1.2)	65 (2.1)	5 (0.2)
Hydrotherapy	0 (0.0)	0 (0.0)	0 (0.0)
**Pharmacologic agents**			
***Patients with* ≥ *1 therapy, n (%)***	3,326 (56.0)	1,719 (55.8)	1,607 (56.1)
*Antiepileptic drugs*	3,258 (54.8)	1,703 (55.3)	1,555 (54.3)
Other antiepileptics	1,763 (29.7)	973 (31.6)	790 (27.6)
Levetiracetam	1,305 (22.0)	798 (25.9)	507 (17.7)
Diazepam	1,177 (19.8)	865 (28.1)	312 (10.9)
Clonazepam	906 (15.3)	534 (17.3)	372 (13.0)
Lamotrigine	664 (11.2)	298 (9.7)	366 (12.8)
Clobazam	572 (9.6)	395 (12.8)	177 (6.2)
Divalproex	514 (8.7)	239 (7.8)	275 (9.6)
Topiramate	493 (8.3)	258 (8.4)	235 (8.2)
Carbamazepine	344 (5.8)	47 (1.5)	297 (10.4)
Valproate	11 (0.2)	3 (0.1)	8 (0.3)
*Sedative/hypnotics*	407 (6.9)	156 (5.1)	251 (8.8)
Phenobarbital	177 (3.0)	49 (1.6)	128 (4.5)
Midazolam	135 (2.3)	96 (3.1)	39 (1.4)
Zolpidem	49 (0.8)	6 (0.2)	43 (1.5)
Triazolam	38 (0.6)	3 (0.1)	35 (1.2)
Other sedatives	27 (0.5)	8 (0.3)	19 (0.7)
Zaleplon	2 (0.0)	1 (0.0)	1 (0.0)
*Prokinetic agents*	74 (1.2)	17 (0.6)	57 (2.0)
Metoclopramide	74 (1.2)	17 (0.6)	57 (2.0)
Other prokinetic agents	0 (0.0)	0 (0.0)	0 (0.0)
*Nutritional supplements*	32 (0.5)	12 (0.4)	20 (0.7)
Other nutritional supplements	25 (0.4)	9 (0.3)	16 (0.6)
Levocarnitine	8 (0.1)	3 (0.1)	5 (0.2)
*Antiarrhythmic drugs*	4 (0.1)	3 (0.1)	1 (0.0)

*Abbreviations*: *RTT* Rett syndrome, *SD* Standard deviation

^a^The observation period was defined as the period from the index date (i.e., date of the first observed medical claim with a diagnosis of RTT) to the earliest of end of clinical activity or end of data availability (i.e., October 31, 2019)

**Fig. 3:**
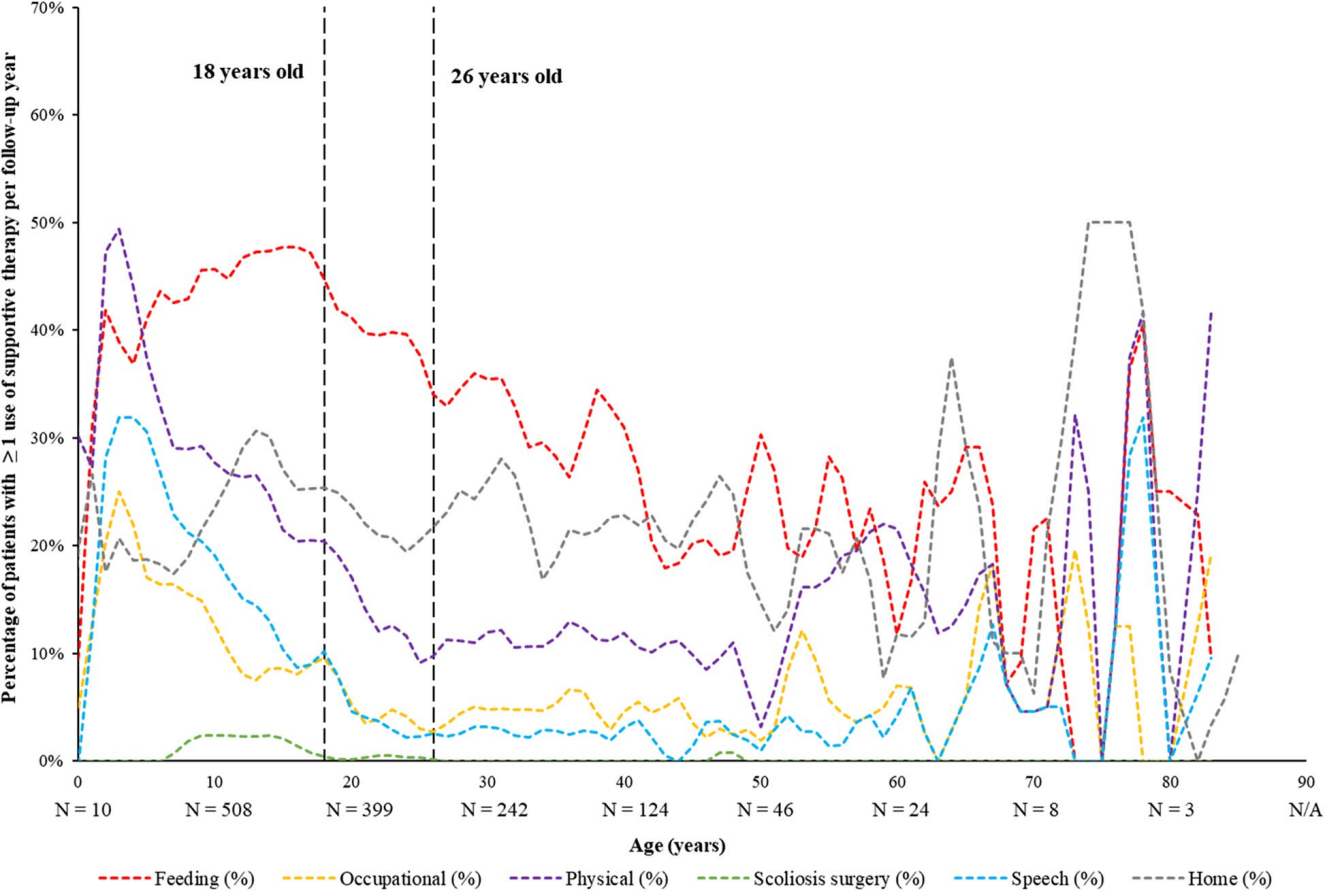
Percentage of Patients with Yearly Supportive Therapies by Age During the Observation Period, All Patients

Based on panel plot data, use of physical, speech-language, and occupational therapies was highest during early childhood (3–4 years of age), before decreasing in use by over 50% by 18 years of age (Fig. 3). Use of feeding assistance grew over childhood and was most prevalent during adolescence (12–17 years of age), while use of home/hospice care remained relatively stable across all ages.

**Fig. 4:**
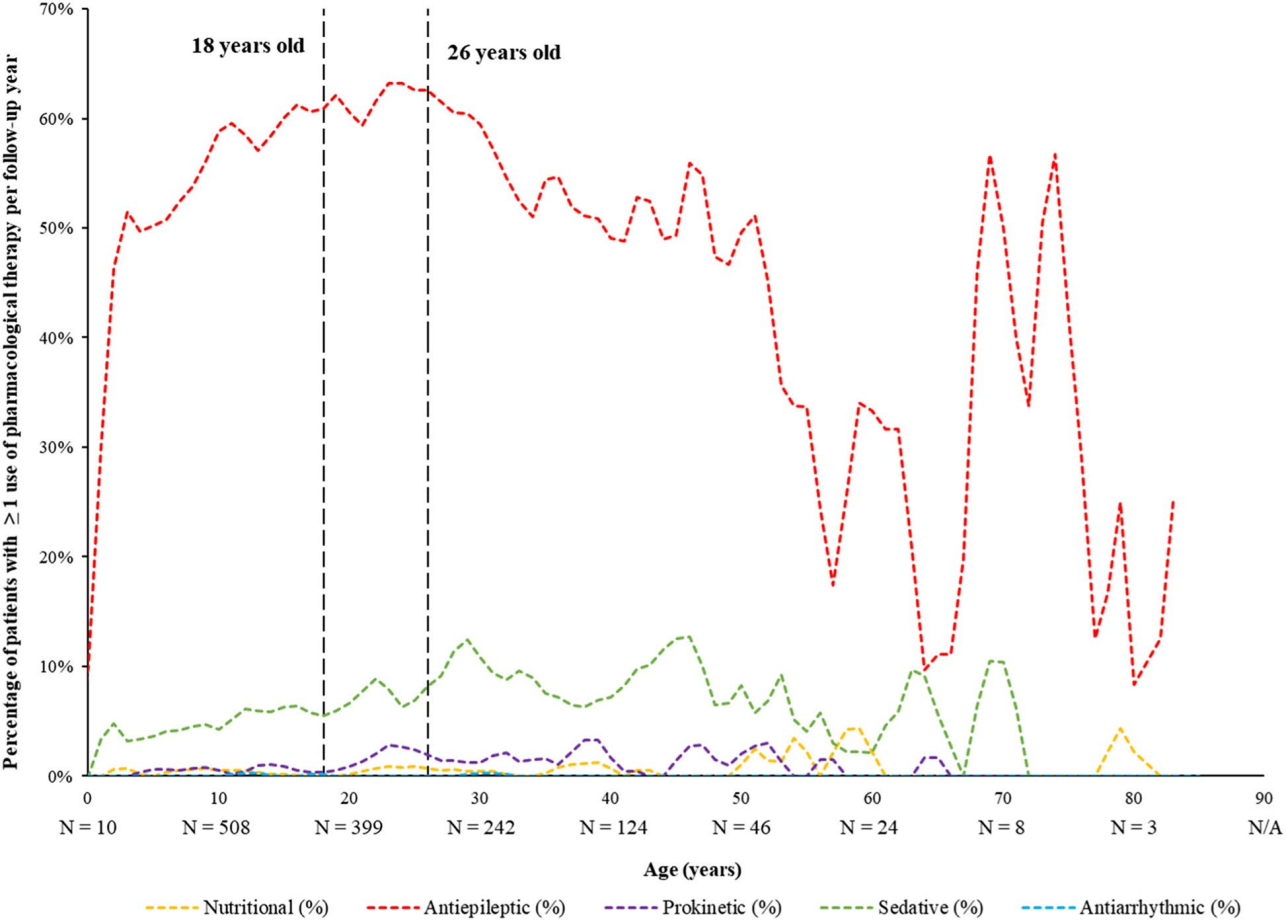
Percentage of Patients with Yearly Pharmacological Therapies by Age During the Observation Period, All Patients

Regarding use of pharmacologic agents, antiepileptic use was highest between 24–26 years of age and sustained into later adulthood, while use of sedatives and prokinetic agents were generally low but rose in early adulthood (≥ 26 years of age; Fig. 4).

## Discussion

This retrospective, real-world study used healthcare claims data to evaluate the healthcare journey of patients with RTT and provide new insights into the disease burden with respect to clinical manifestations, HRU and costs, as well as treatment patterns. Notably, patients with RTT had substantial concomitant disease burden across their lifespan, as evidenced by the high prevalence and rates of clinical manifestations, as well as reliance on both pharmacologic and supportive therapy. Moreover, patients with RTT incurred a significant HRU burden, with nearly one healthcare visit per week following RTT diagnosis (i.e., 44.43 PPPY) and nearly half of all visits and corresponding costs attributed to RTT. As a descriptive comparison with other neurological conditions, children and adults with spinal muscular atrophy type 1 were found to have 59.4 days with medical visits PPPY (including inpatient, ED, and OP) in one study [11], while female adults with cerebral palsy had 38.3 annual medical visits (including inpatient, OP and other, ED, office, and ancillary) in 2016 in a separate study [12]. Therefore, the HRU burden of RTT appears to be in line with that of other debilitating neurological conditions, though the different HRU categories included in these studies may limit comparability. Taken together, these findings contextualize the burden of RTT in the US.

To our knowledge, this is one of the first real-world evidence studies examining the epidemiology and journey of patients with RTT using large administrative claims data from the US. A prior study using a population-based registry from Texas estimated a RTT prevalence of 0.44 per 10,000 females aged 2–18 years [13]; while not directly comparable to our overall prevalence estimates due to the age stratification, our estimates are within range of the registry-based prevalence. However, there is sparse literature evaluating HRU and costs associated with RTT [9]. In a survey-based study of 399 individuals with RTT (95.5% female and 82.2% from the US), 21.4% experienced a hospital admission for LRTI over the previous 5 years, with 11.6% having two or more admissions [10]. Additionally, decreasing ability to walk was associated with higher mean admission rates and longer lengths of hospital stays, while patients who used enteral feeding also had an increased risk of LRTI-related hospital admission [10]. While not directly comparable to the survey findings, feeding assistance and physical therapy were among the most used supportive therapies in the current study, the need for which may have contributed to the large HRU burden of these patients. However, further study is warranted to identify clinical factors and RTT manifestations that may prompt the use of various healthcare resources.

Although the economic burden of RTT has not been well-characterized in the literature, the current study found that female patients with RTT each incurred more than $40,000 per year in healthcare costs, including more than $45,000 per year among pediatric patients and nearly $35,000 among adult patients. As a descriptive contrast, these annual healthcare costs appear to be higher than those associated with other neurologic conditions, such as fragile X syndrome ($17,878 among one female population; 2012 USD) [14], cerebral palsy ($25,844 among one adult female population; 2016 USD) [12], and epilepsy ($15,414 among one adult population; unspecified USD) [15], though the use of different study designs and populations may limit comparability. In the context of other genetic conditions, a study of hospital admissions in a US children’s hospital found that 71% of admitted children had an underlying genetic disorder (including RTT), and 81% of the total yearly hospital charges were accounted for by conditions with a genetic determinant [16], highlighting the particularly large burden associated with genetic disorders like RTT.

While prevalence of clinical manifestations and high rates of HRU persisted into adulthood in this study, outcomes were generally most frequent during childhood. This finding is aligned with the stages of progression of RTT, where rapid regression of acquired motor skills and communication primarily occurs from ages 1–4 years before plateauing through adolescence and young adulthood [4]. Two studies using population-based registries also identified higher use of healthcare services, particularly hospital admissions, among younger patients with RTT, albeit in Australia [17, 18]. Taken together, the larger disease and economic burden of RTT in childhood emphasizes the importance of early diagnosis and treatment to potentially mitigate downstream effects.

Despite the fact that symptoms of RTT typically appear after the first 6–18 months of life [4], many patients may not receive a clinical diagnosis up to 2–4 years later due to diagnostic challenges [6]. While diagnostic delay was not measured in this study, 16.2% of patients received a differential diagnosis prior to their first observed RTT diagnosis, suggesting that the correct diagnosis of RTT was not always given. This observation is in line with prior reports detailing the diagnostic delay that patients and their families often face when seeking an initial diagnosis [6, 19, 20]. For instance, in a longitudinal study of RTT patients from multiple US sites, the median age of diagnosis was 2.7–3.8 years, but most patients already exhibited the core manifestations of RTT prior to diagnosis, with several characteristic features present for over a year beforehand [6]. Furthermore, despite the important role of pediatricians in the identification of developmental disorders, only a minority of RTT cases (5.2%) were diagnosed by pediatricians, with most cases being diagnosed by subspecialists like neurologists [6]. This suggests that RTT is a more complex developmental disorder that may require treatment from medical providers with specialized training and experience. These findings signal the need for greater awareness of the complex nature and varied manifestations of RTT. Indeed, earlier diagnosis of RTT is associated with many benefits, including reduced psychosocial stress during the search for a diagnosis, earlier opportunities for specific intervention and counselling, and earlier surveillance of symptoms to slow disease progression [6, 19]. Interestingly, frequency of *MECP2* genetic testing prior to the first observed medical claim with a diagnosis of RTT in the present study was low. While reasons for this observation are unknown, it is possible that genetic testing was indeed conducted but not captured in the claims data due to factors such as lack of coverage by the health insurance plan or an out-of-network claim. Further research is warranted to confirm *MECP2* genetic testing frequency among patients with RTT in the US.

The large clinical and economic burden of RTT observed in this study throughout patients’ lifespans suggests a substantial unmet need for treatment options that may modify the disease course and progression of RTT rather than managing its symptoms. Additionally, there is a lack of standardization and quality metrics for currently available therapies to ensure the best outcomes for patients with RTT [3]. As such, further research is warranted to establish a standard of care for patients with RTT and develop targeted treatment options that may improve quality of life and functional outcomes for this debilitating condition.

### Limitations

The findings of this study should be interpreted in light of some limitations. Patient enrollment data were not available in the IQVIA Dx and LRx databases; as such, continuous enrollment could not be established, which could introduce additional uncertainty in the estimation of endpoints of interest. For instance, annual prevalence was potentially underestimated since only patients who accessed health care services (medical or pharmacy) at least once per year during the study period were captured. Annual incidence, particularly in the adult age groups, was also subject to risk of overestimation since diagnoses of RTT are often made during early childhood but may not have been captured in the medical claims data for patients who were only included in the IQVIA Dx database as an adult; therefore, these patients may have been misclassified as having incident RTT in adulthood. Relatedly, the index diagnosis of RTT was the first diagnosis observed in the data, and thus does not necessarily reflect the first diagnosis that the patient has ever received.

This analysis relied on pre-adjudicated medical claims from IQVIA Dx database, for which payment amounts have not yet been finalized and are often inflated compared to their adjudicated equivalent. Therefore, healthcare costs may be overestimated. In addition, diagnosis and procedure codes associated with medical claims were used to determine the presence of important clinical characteristics; therefore, misspecification of these codes may have resulted in misclassification of patients and endpoints of interest. For instance, diagnoses for symptoms may have been coded to RTT rather than the individual symptom. However, due to the rare nature of RTT, it is likely that any use of the ICD-10-CM diagnosis code was for the purpose of associating the healthcare service with a diagnosis of RTT. Furthermore, missing information is often a potential issue in retrospective analyses of claims data that can result in measurement error. For example, overall coverage of institutional claims is lower than that of OP claims in the IQVIA Dx database, potentially resulting in incomplete capture of inpatient, ED, and long-term care/skilled nursing facility visits. Lastly, as analyses from this study were primarily conducted in female patients with RTT, our study findings may not be generalizable to male patients with RTT, who may have a differential disease burden. Despite these limitations, the IQVIA Dx and LRx databases are an ideal data source for this analysis since information is reported from various health care providers (e.g., doctors, pharmacies, and hospitals) with which a patient may interact, regardless of health care system, insurance type (including cash-paying patients with no insurance), or location.

## Conclusions

Findings from this real-world study highlight the substantial disease burden experienced by patients with RTT, including a high prevalence of clinical manifestations, high rates of HRU and annual healthcare costs, as well as a reliance on pharmacologic and supportive therapies. These findings underscore the unmet need for effective therapies to manage the multifactorial manifestations of RTT and restore function to activities of daily life for patients. Future studies could provide additional insight by investigating the humanistic burden associated with RTT, including the change in quality of life with continuing disease.

## Data Availability

The data that support the findings of this study are available from IQVIA™ but restrictions apply to the availability of these data, which were used under license for the current study, and therefore, are not publicly available. Data are however available from the authors upon reasonable request and with permission from IQVIA™.

## Abbreviations

ED: Emergency department
HRU: Healthcare resource utilization
ICD-10-CM: International Classification of Diseases, 10^th^ Revision, Clinical Modification
LRTI: Lower respiratory tract infection
NIH: National Institutes of Health
OP: Outpatient
PPPY: Per-patient-per-year
RTT: Rett syndrome
SD: Standard deviation
US: United States
USD: United States dollar
